# Systematic analysis of the test design and performance of AI/ML-based medical devices approved for triage/detection/diagnosis in the USA and Japan

**DOI:** 10.1038/s41598-022-21426-7

**Published:** 2022-10-07

**Authors:** Mitsuru Yuba, Kiyotaka Iwasaki

**Affiliations:** 1 Cooperative Major in Advanced Biomedical Sciences, Joint Graduate School of Tokyo Women’s Medical University and Waseda University, Waseda University, 2-2 Wakamatsucho, Shinjuku, Tokyo, 162-8480 Japan; 2 Department of Modern Mechanical Engineering, School of Creative Science and Engineering, Waseda University, Tokyo, Japan; 3 Department of Integrative Bioscience and Biomedical Engineering, Graduate School of Advanced Science and Engineering, Waseda University, Tokyo, Japan; 4 Institute for Medical Regulatory Science, Waseda University, Tokyo, Japan

**Keywords:** Health care, Health policy

## Abstract

The development of computer-aided detection (CAD) using artificial intelligence (AI) and machine learning (ML) is rapidly evolving. Submission of AI/ML-based CAD devices for regulatory approval requires information about clinical trial design and performance criteria, but the requirements vary between countries. This study compares the requirements for AI/ML-based CAD devices approved by the US Food and Drug Administration (FDA) and the Pharmaceuticals and Medical Devices Agency (PMDA) in Japan. A list of 45 FDA-approved and 12 PMDA-approved AI/ML-based CAD devices was compiled. In the USA, devices classified as computer-aided simple triage were approved based on standalone software testing, whereas devices classified as computer-aided detection/diagnosis were approved based on reader study testing. In Japan, however, there was no clear distinction between evaluation methods according to the category. In the USA, a prospective randomized controlled trial was conducted for AI/ML-based CAD devices used for the detection of colorectal polyps, whereas in Japan, such devices were approved based on standalone software testing. This study indicated that the different viewpoints of AI/ML-based CAD in the two countries influenced the selection of different evaluation methods. This study’s findings may be useful for defining a unified global development and approval standard for AI/ML-based CAD.

## Introduction

Software development for medical devices utilizing artificial intelligence (AI) and machine learning (ML) has been evolving rapidly. The amount of AI/ML-based software used in medical devices approved by the US Food and Drug Administration (FDA) has been increasing every year^1,2^. An annual survey by the American College of Radiology showed that more than 30% of radiologists used AI to improve diagnostic interpretation accuracy^3^. The use of AI/ML for computer-aided detection (CADe), computer-aided diagnosis (CADx), and computer-aided simple triage (CAST^4^) has allowed practitioners to use these computer-aided methods to their full potential. However, owing to the rapid progress of AI/ML-based CAD, there is a demand to properly evaluate the efficacy and safety of the methods used to acquire approval^5–7^.

Evaluation methods for AI/ML-based CAD devices can be classified into standalone software testing and reader study testing. Standalone software testing is defined as a performance test of the AI-only using test data that are collected retrospectively. The advantage is cost and time savings because there is no need to recruit readers for performance evaluation. However, because it cannot be used to evaluate performance in clinical practice, it cannot evaluate usability and the affection of AI assistance. Reader study testing is defined as a performance test that evaluates the interaction of AI with physicians on diagnostic or detection accuracy. It is necessary to recruit readers for testing, which is more costly and time-consuming than standalone software testing. Reader study testing can be performed not only prospectively but also retrospectively using previously collected images.

A recent prospective randomized controlled trial (RCT) for evaluating the performance of AI/ML-based CAD for cataract detection failed to demonstrate diagnostic accuracy comparable to that of the pilot study. This study indicated the need to evaluate the influence of physician intervention in clinical practice^8^. The most recent reader study testing^9,10^, in which 45 clinicians from 9 clinical institutions participated in the evaluation of a product intended to detect breast cancer, compared the results with and without AI assistance and reported that AI assistance improved clinicians’ accuracy. Hence, evaluations differ depending on the test design and domain. Therefore, it would be beneficial to developers of AI/ML-based CAD to know whether the criteria for diagnostic accuracy can be evaluated by standalone software testing or should be evaluated by reader study testing that includes the influence of physicians on diagnostic accuracy.

In this study, we investigate the AI/ML-based CAD devices approved by the FDA and the Pharmaceuticals and Medical Devices Agency (PMDA) in Japan and analyzed their requirements in terms of target and study design to provide insights into the global development of AI/ML-based CAD.

## Results

### AI/ML-based medical devices in the USA

**Figure 1:**
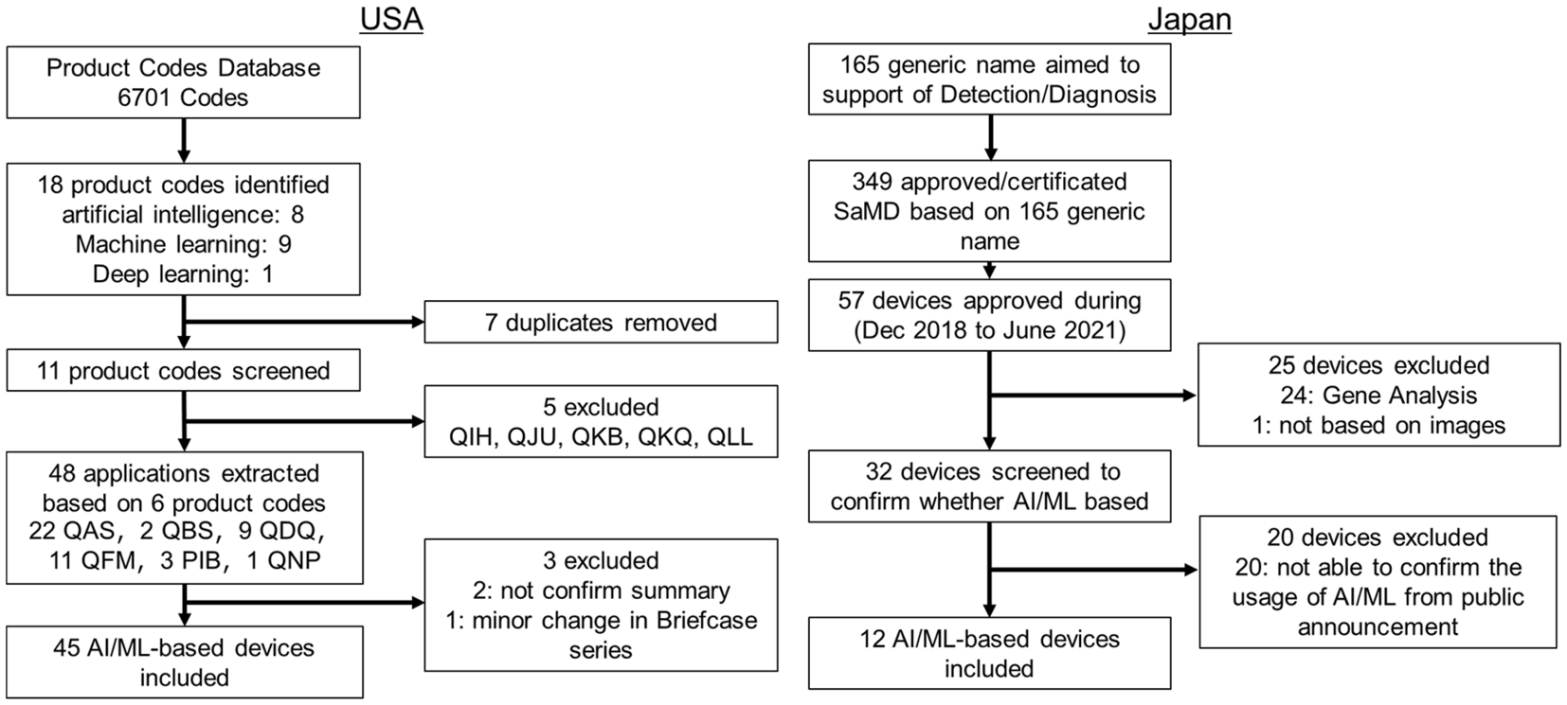
Flowchart for extraction of AI/ML-based CAD devices approved in the USA and Japan.

**Figure 2:**
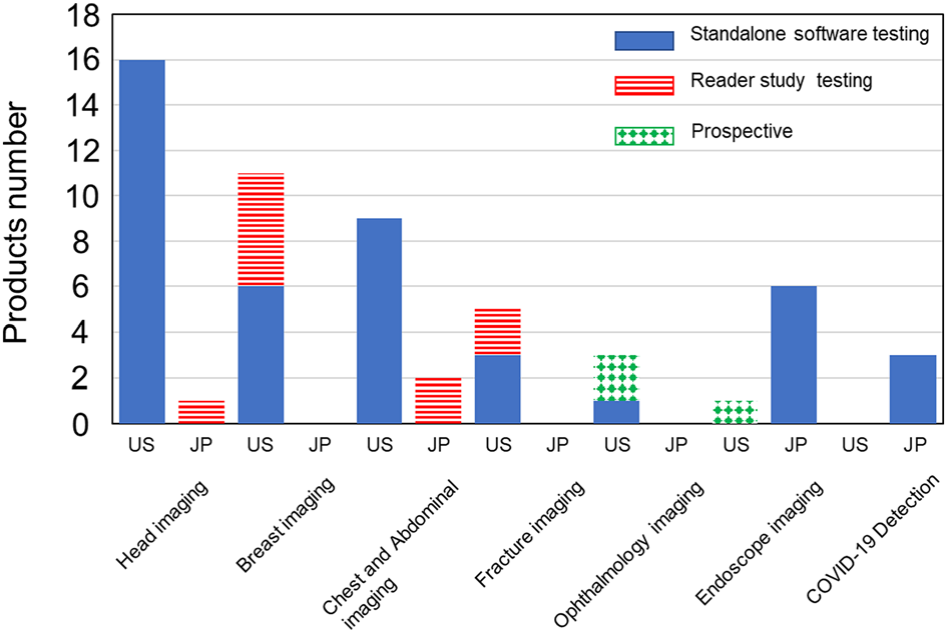
Number of approved AI/ML-based CAD devices in the USA and Japan.

We identified 45 FDA-approved AI/ML-based medical devices using the FDA Product Code Classification Database^11^ (Fig. 1). There were 45 devices for a variety of targets categorized as follows: 16 (35.6%) for triage intracranial hemorrhage or large vessel occlusion detection, 11 (24.4%) for breast cancer detection/diagnosis, 9 (20.0%) for triage pulmonary embolism, pneumothorax, pleural effusion, or intra-abdominal free gas, 5 (11.1%) for wrist fracture, cervical spine bone fracture, vertebral compression fracture, or rib fracture diagnosis, 3 (6.7%) for diabetic retinopathy diagnosis, and 1 (2.2%) for colorectal polyp detection. In terms of the study design of the 45 devices, 35 (77.8%) were approved based on standalone software testing (Table 1). The other 10 (22.2%) devices were approved based on reader study testing (Table 2). In terms of sources of clinical data, three studies (6.7%) were conducted prospectively, while 42 (93.3%) studies used previously collected clinical data to evaluate efficacy (Fig. 2).

**Table 1:** Characteristics of the 35 FDA approved AI/ML-based CAD evaluated by standalone software testing.

Device	Intended use/summary	CAD	Imaging modality	Test Case	Sensitivity/specificity	AUC	Approved pathway	Approved date	Manufacture
**Chest and abdomen imaging**									
BriefCase forPulmonary Embolism triage	Triage and notification of pulmonary embolism	CAST	CT	184	90.6/89.9		510(k)	April 2019	Aidoc Medical Ltd
HealthPNX	Triage and notification of pneumothorax	CAST	X-ray	588	93.1/92.9	0.98	510(k)	May 2019	Zebra Medical Vision Ltd
Critical Care Suite	Triage and notification of pneumothorax	CAST	X-ray	804	84.3/93.5	0.96	510(k)	August 2019	GE Medical Systems LLC
HealthCXR	Triage and notification of pleural effusion	CAST	X-ray	554	96.7/93.1	0.98	510(k)	November 2019	Zebra Medical Vision Ltd
Red Dot	Triage and notification of pneumothorax	CAST	X-ray	888	94.6/87.9	0.97	510(k)	February 2020	Behold. AI Technologies Limited
AIMI-Triage CXR PTX	Triage and notification of pneumothorax	CAST	X-ray	300	92/90	0.96	510(k)	April 2020	RADLogics Inc
BriefCase for Intra-abdominal Free Gas triage	Triage and notification of intra-abdominal free gas	CAST	CT	184	91/88.9		510(k)	June 2020	Aidoc Medical Ltd
BriefCase for incidental Pulmonary Embolism triage	Triage and notification of incidental pulmonary embolism	CAST	CT	268	90.5/88.7		510(k)	August 2020	Aidoc Medical Ltd
CINA CHEST	Triage and notification of pulmonary embolism (PE) and aortic dissection (AD)	CAST	Chest CTA/thoraco-abdominal CTA	PE: 396 AD: 298	91.1/91/8 96.4/97.5		510(k)	May 2021	Avicenna.AIt
**Head imaging**									
ContaCT	Triage and notification of large vessel occlusion	CAST	CTA	300	87.8/89.6	0.91	de novo	February 2018	Viz.AI Inc
BriefCase for Intracranial Hemorrhage triage	Triage and notification of intracranial hemorrhage	CAST	CT	198	93.6/92.3		510(k)	August 2018	Aidoc Medical Ltd
Accipiolx	Triage and notification of intracranial hemorrhage	CAST	CT	360	92/86		510(k)	October 2018	MaxQ-AI Ltd
HealthICH	Triage and notification of intracranial hemorrhage	CAST	CT	427	94.4/92.5		510(k)	June 2019	Zebra Medical Vision Ltd
DeepCT	Triage and notification of intracranial hemorrhage	CAST	CT	260	93.8/92.3		510(k)	July 2019	Deep01 Limited
BriefCase for Large Vessel Occlusion triage	Triage and notification of large vessel occlusion	CAST	CTA	383	88.8/87.2		510(k)	December 2019	Aidoc Medical Ltd
Viz ICH	Triage and notification of intracranial hemorrhage	CAST	CT	261	93/90	0.96	510(k)	March 2020	Viz ai Inc
Rapid ICH	Triage and notification of intracranial hemorrhage	CAST	CT	336	89.9/94.3		510(k)	March 2020	iSchemaView Inc
CuraRad-ICH	Triage and notification of intracranial hemorrhage	CAST	CT	388	90.6/93.1		510(k)	April 2020	CuraCloud Corp
NineAI	Triage and notification of intracranial hemorrhage (ICH) and mass effect (ME)	CAST	CT		ICH:89.9/97.4 ME:96.4/91.1		510(k)	April 2020	Nines Inc
qER	Triage and notification of intracranial hemorrhage (ICH), mass effect (ME), midline shift (MS) and cranial fracture (CF)	CAST	CT	Total:1320 ICH:629 ME:471 MS:414 CF:248	98.5/91.2 96.9/93/9 96.3/96 97.3/95.3 96.7/92.7	0.98 0.99 0.99 0.97	510(k)	June 2020	Qure. ai Technologies
CINA	Triage and notification of intracranial hemorrhage (ICH) and large vessel occlusion (LVO)	CAST	CT/CTA	ICH:814 LVO:476	91.4/97.5 97.9/97.6	0.94 0.98	510(k)	June 2020	AVICENNA. AI
Rapid LVO	Triage and notification of large vessel occlusion	CAST	CTA		97/95.6	0.99	510(k)	July 2020	iSchemaView Inc
Accipiolx	Improve performance by changing algorithm	CAST	CT	360	97/93	N/A	510(k)	August 2020	MaxQ AI Ltd
HALO	Triage and notification of large vessel occlusion	CAST	CTA	364	91.1/87	0.97	510(k)	November 2020	NiCo-Lab BV
Viz ICH	Addition of GE’s non-contrast CT as supported systems	CAST	CT	387	95/96	0.97	510(k)	March 2021	Viz ai Inc
**Fracture imaging**									
BriefCase for C-Spine fracture triage	Triage and notification of cervical spine bone fracture	CAST	CT	186	91.7/88.6		510(k)	May 2019	Aidoc Medical Ltd
HealthVCF	Triage and notification of vertebral compression fractures	CAST	CT	611	90.2/86.9	0.95	510(k)	May 2020	Zebra Medical Vision Ltd
uAI Easy Triage-Rib	Triage and notification of multiple (3 or more) acute rib fracture	CAST	CT	200	92.7/84.7	0.93	510(k)	January 2021	Shanghai United Imaging Intelligence Co., Ltd
**Breast imaging**									
cmTriage	Triage and notification of breast cancer	CAST	Mammogram	1255	86.9/88.5	0.95	510(k)	March 2019	CureMetrix Inc
ProFound AI Software V2.1	Application to add Siemens Modalities as supported systems	CADe/ CADx	Digital breast tomosynthesis	694			510(k)	October 2019	iCAD Inc
Transpara V1.5	Addition of Fujifilm’s mammogram as supported systems	CADe/ CADx	Mammogram				510(k)	December 2019	ScreenPoint Medical BV
HealthMammo	Triage and notification of breast cancer	CAST	Mammogram	835	89.9/90.7	0.96	510(k)	July 2020	Zebra Medical Vision Ltd
Saige-Q	Triage and notification of breast cancer	CAST	Mammogram/Digital breast tomosynthesis	Mammogram: 1333 DBT: 1528	92.2/91.2 98.3/95.7	0.96 0.98	510(k)	April 2021	DeepHealth, Inc
Transpara V1.7	Addition of Fujifilm’s digital breast tomosynthesis as supported systems	CADe/ CADx	Mammogram/Digital breast tomosynthesis				510(k)	June 2021	ScreenPoint Medical B.V
**Ophthalmology imaging**									
IDx-DR	Addition of Training mode, and change the user interface	CADe/ CADx	Fundus camera				510(k)	June 2021	Digital Diagnostics Inc

**Table 2:** Characteristics of the 10 FDA approved AI/ML-based CAD evaluated by reader study testing.

Device	Intended use/summary	CAD	Imaging modality	Study design	Test case	Reader	Sensitivity/specificity	AUC	Approved pathway	Approved date	Manufacture
**Endoscope imaging**											
GI Genius	Detection of colonic mucosal lesions	CADe	Endoscopy	Prospective, RCT	685	6	ADR: 54.8 (40.4)		de novo	April 2021	Cosmo Artificial Intelligence—AI, LTD
**Ophthalmology imaging**											
IDx-DR	Detection and diagnosis of more than mild diabetic retinopathy	CADe/CADx	Fundus camera	Prospective	900		87/90		de novo	April 2018	IDx LLC
EyeArt	Detection and diagnosis of more than mild diabetic retinopathy and vision-threatening diabetic retinopathy	CADe/CADx	Fundus camera	Prospective	942		95.5/86.5		510(k)	August 2020	Eyenuk Inc
**Fracture imaging**											
OsteoDetect	Detection and diagnosis of distal radius fractures of adult wrists	CADe	X-ray	Retrospective, MRMC, Fully-crossed	200	24	80/91 (74/88)	0.88 (0.84)	de novo	May 2018	Imagen Technologies Inc
FractureDetect	Detection and diagnosis of 12 fractures (ankle, clavicle, elbow, femur, forearm, hip, humerus, knee, pelvis, shoulder, tibia/fibula, wrist)	CADe	X-ray	Retrospective, MRMC, Fully-crossed	175	24	90/91.8 (82/89)	0.95 (0.91)	510(k)	July 2020	Imagen Technologies Inc
**Breast imaging**											
Transpara V1.3	Detection and diagnosis of breast cancer	CADe/CADx	Mammogram	Retrospective, MRMC, Fully-crossed	240	14		0.88 (0.86)	510(k)	November 2018	ScreenPoint Medical BV
ProFound AI Software V2.0	Detection and diagnosis of breast cancer	CADe/CADx	Digital breast tomosynthesis	Retrospective, MRMC, Fully-crossed	260	24	85/69 (77/62)	0.85 (0.79)	510(k)	December 2018	iCAD Inc
Transpara V1.6	Addiction of digital breast tomosynthesis as supported systems	CADe/CADx	Mammogram/ Digital breast tomosynthesis	Retrospective, MRMC, Fully-crossed	240	18		0.86 (0.83)	510(k)	March 2020	ScreenPoint Medical BV
MammoScreen	Detection and diagnosis of breast cancer	CADe/CADx	Mammogram	Retrospective, MRMC	240	14		0.8 (0.77)	510(k)	March 2020	Therapixel
Genius AI Detection	Detection and diagnosis of breast cancer	CADe/CADx	Digital breast tomosynthesis	Retrospective, MRMC	390	17	75.9/25.8 (66.8/23.4)	0.82 (0.79)	510(k)	November 2020	Hologic Inc

Sensitivity/specificity and AUC are shown as with AI (without AI).

### AI/ML-based medical devices in Japan

We identified 12 PMDA-approved AI/ML-based medical devices using the database of the Japan Association for the Advancement of Medical Equipment Search (JAAME Search)^12^ (Fig. 1). The target of the devices was as follows: 6 (50%) for colorectal lesion detection, 3 (25%) for detection of covid-19 infection, 2 (16.7%) for detection of pulmonary nodules, and 1 (8.3%) for cerebral aneurysm detection. In terms of the study design of the 12 devices, 9 (75%) were approved based on standalone software testing (Table 3), and three (25%) were approved based on reader study testing (Table 4). No prospective studies have been conducted to acquire market approval (Fig. 2).

**Table 3:** Characteristics of the 9 PMDA approved AI/ML-based CAD evaluated by standalone software testing.

Device	Intended use/summary	CAD	Imaging modality	Test case	Sensitivity/specificity	AUC	Accuracy	Approved date	Manufacture
**Endoscope imaging**									
EndoBRAIN	Diagnosis of neoplastic or non-neoplastic	CADx	Endocytoscopy	100	96.9		98	December 2018	CYBERNET
EndoBRAIN-UC	Diagnosis of inflammation for Ulcerative colitis	CADx	Endocytoscopy	1000	95.1/90.7		91.9	April 2020	CYBERNET
EndoBRAIN-EYE	Detection of colonic mucosal lesions	CADe	Endoscopy	300	96.3/93.7			January 2020	CYBERNET
EndoBRAIN-Plus	Diagnosis of non-neoplastic, adenoma or invasive cancer	CADx	Endocytoscopy	50	91.8/97.3			July 2020	CYBERNET
EW10-EC02	Detection and diagnosis of colonic mucosal lesions	CADe/CADx	Endoscopy	WLI: 912 LCI: 943 BLI: 296 WLI: 308	94.5 96		94.9 93.2	September 2020	FUJIFILM
WISE VISION	Detection of colonic mucosal lesions	CADe	Endoscopy	350	83/89			November 2020	NEC
**COVID-19 detection**									
InferRead CT Pneumonia	Detection of pneumonia cause by covid-19	CADe	CT	190	77.1/90.7			June 2020	CES decartes
Ali-M3	Detection of pneumonia cause by covid-19	CADe	CT	704	89.6/37.1			June 2020	MIC Medical Corp
FS-AI693	Detection of pneumonia cause by covid-19	CADe	CT	217	87.5/37.1	0.74		May 2021	FUJIFILM

**Table 4:** Characteristics of the 3 PMDA approved AI/ML-based CAD evaluated by Reader Study Testing.

Device	Intended use/summary	CAD	Imaging modality	Test case	Reader	Sensitivity/specificity	AUC	Accuracy	Approved date	Manufacture
**Chest and abdomen imaging**										
FS-AI688	Detection of lung nodule	CADe	CT	36	10	61.4 (49)			May 2020	FUJIFILM
EIRL X-Ray Lung nodule	Detection of lung nodule	CADe	CT	320	18	56.9/96.7 (45.4/96.3)	0.76 (0.70)	88.4 (85.6)	August 2020	LPIXEL
**Head imaging**										
EIRL aneurysm	Detection of Unruptured cerebral aneurysm	CADe	MRA	200	20	77.2/72.1 (68.2/79.4)		0.75 (0.71)	September 2019	LPIXEL

Sensitivity/specificity and AUC are shown as with AI (without AI).

### Endoscope imaging

As a targeting device for colorectal lesions, GI Genius (Medtronic) was approved by the FDA based on the data of a prospective RCT. A total of 685 patients were enrolled and divided into two groups. The adenoma detection rate (ADR) was compared between participants diagnosed using traditional endoscopy methods and those diagnosed using CADe. The efficacy of CAD was demonstrated by the fact that the detection rate by CADe exceeded that of traditional endoscopy methods without CADe (54.8% vs. 40.4%)^13^.

In Japan, six devices^14–19^ were approved for colorectal lesions, and all devices were evaluated using retrospective data and standalone software testing. Of the six devices, three were used for analyzing images captured with an endocytoscope (1 for differentiating the degree of inflammation of ulcerative colitis, and 2 for differentiating the degree of tumor malignancy), and 3 were used for analyzing images captured by the endoscope.

The average number of images used for the evaluation of the devices designed for endocytoscope was 383.3 (minimum 50, maximum 1000). The average sensitivity, specificity, and accuracy rates were 94.6% (minimum 91.8, maximum 96.9), 94.1% (minimum 91, maximum 97.3), and 95% (minimum 92, maximum 98). AUC was not reported.

For devices designed for endoscopic detection of polyps, video data were used instead of images for performance evaluation. The efficacy of EndoBRAIN-EYE (CYBERNET)^16^ was evaluated using 12 h of videos including 300 lesions. The efficacy of WISE VISION (NEC)^18^ was evaluated using videos including 350 lesions, and the number of continuous frames in which the lesions were identified was the index of performance. EW1-EC02 (FUJIFILM)^19^ has both CADe and CADx functions. The CADe performance was evaluated based on the successful continuous detection of polyps. The CADx performance was evaluated based on the correct identification of a lesion as a tumor or non-tumor lesion^20^.

### Chest and abdominal imaging

In the USA, nine medical devices^21–29^ aimed at triaging pulmonary embolism, pneumothorax, pleural effusion, or intra-abdominal free gas, all categorized as CAST, were approved through evaluation of standalone software testing. An average of 496 images (minimum 184, maximum 888) were used for performance evaluation. The sensitivity and specificity reported by all nine studies had averages of 92.0% (minimum 84.3, maximum 96.4) and 91.4% (minimum 87.9, maximum 97.5). AUC was only reported by 5 studies and the average was 0.97 (minimum 0.96, maximum 0.98).

In Japan, two devices^30,31^ for lung nodule detection were categorized as CADe and approved based on reading study testing. An average of 178 images (minimum 36, maximum 320) were used for testing. The reported averages of sensitivity and specificity were 59.1% (minimum 56.9, maximum 61.4) and 63.9% (minimum 37.1, maximum 90.7), respectively. AUC was not reported by either study.

### Head imaging

In the USA, 16 devices^32–47^ designed for the triage of intercranial hemorrhage and/or large vessel occlusion were labeled as CAST devices and approved after analysis of performance evaluation results. An average of 414.6 images (minimum 198, maximum 1320) were used for evaluation. The averages of the sensitivity and specificity reported by all studies were 93.6 (minimum 87.7, maximum 98.5) and 92.8 (minimum 86, maximum 97.6), respectively. The average AUC (reported by only 7 studies) was 0.97 (minimum 0.91, maximum 0.99).

The Accipilox (MaxQ AI Ltd.), a medical device targeting the detection of intercranial hemorrhage, was first approved by the FDA in 2018^34^. However, after changing the original algorithm from an ML-based to a convolutional neural network (CNN)-based algorithm in 2020^45^, application for re-approval became necessary. With this change, the sensitivity increased from 92 to 97%, and the specificity increased from 86 to 93%. Both tests were performed using 360 images. Similarly, the Viz ICH (Viz ai Inc.)^38,47^, another device for intracranial hemorrhage detection, was granted FDA clearance after the development of an add-on allowing for AI automatic detection on non-contrast CT scans acquired on scanners manufactured by general electric (GE). Device sensitivity increased from 93 to 95%, specificity increased from 90 to 96%, and AUC increased from 0.96 to 0.97.

In Japan, a CADe device that analyzes head magnetic resonance angiography images to detect unruptured cerebral aneurysms^48^ was approved based on reader study testing. A total of 200 images were used for the testing. The reported sensitivity and specificity were 77.2% and 72.1%, respectively. AUC was not reported.

### Ophthalmology imaging

In the USA, two devices^49,50^ for diabetic retinopathy diagnosis were approved using data from a prospective study. The average number of images used for performance evaluation was 921 (minimum 900, maximum 942). Sensitivity and specificity were 91.2% (minimum 87, maximum 95.5) and 88.6% (minimum 86.5, maximum 90), respectively. AUC was not reported. Notably, the percentage of images that could be correctly evaluated through AI was calculated using an imageability factor, and the reported average was 97.3% (minimum 96, maximum 98.6). Both devices were used in primary care facilities in the USA and were developed to help caregivers decide whether to encourage patients to see specialists based on the results of AI analysis.

Regarding IDx-DR (Digital Diagnosis Inc.), a second application for the addition of a training mode and alterations to the user interface were approved^51^. However, additional performance evaluation was not conducted at the time of the second application. AI/ML-based CAD for the diagnosis of diabetic retinopathy has not yet been approved by Japanese agencies.

### Fracture imaging

Of the five devices^52–56^ aimed at fracture detection that received FDA approval, three were evaluated by standalone software testing^54–56^. These three devices were categorized as CAST for cervical spine fracture, vertebral compression fracture, and rib fracture, and CT images were used for analyses. The other two devices were developed for wrist fracture detection^52^ and 12 types of fracture detection on X-ray images^53^. These two devices were approved based on reader study testing and an improvement in diagnostic accuracy using X-ray images was demonstrated with the assistance of the software. Standalone software testing was conducted using an average of 332.3 images (minimum 186, maximum 611). The average sensitivity, specificity, and AUC were 91.5% (minimum 90.2, maximum 92.7), 86.7% (minimum 84.7, maximum 88.6), and 0.94 (minimum 0.93, maximum 0.95), respectively. AUC was only reported for two of the three devices (not reported for the cervical spine fracture triage device).

Reader study testing was performed using an average of 187.5 images (minimum 175, maximum 200). Average sensitivity, specificity, and AUC were 85% (minimum 80, maximum 90), 91.4% (minimum 91, maximum 91.8), and 0.91 (minimum 0.88, maximum 0.95), respectively. AI/ML devices aimed at detecting fractures are yet to be approved in Japan.

### Breast imaging

Among the 11 devices^57–67^ for detection and diagnosis of breast cancer approved in the USA, six were evaluated based on standalone software testing^57,58,60,62–64^. Five devices categorized as CADe/CADx, which were designed to detect suspected breast cancer sites and malignancy levels, were approved based on reader study testing^59,61,65–67^.

Among the devices evaluated through standalone software testing, ProFound AI Software V2.1 (iCAD Inc.)^58^, Transpara V1.5 (ScreenPoint Medical BV)^60^, and Transpara V1.7 (ScreenPoint Medical BV)^62^ were classified as CADe/CADx devices. However, all three devices were upgraded versions of devices that had been approved based on reader study testing, with the addition of mammography or digital breast tomosynthesis. For the Transpara V1.6 (ScreenPoint Medical BV)^61^, a second reader study test was conducted at the time of the upgrade from the previous version because it added digital breast tomosynthesis as usable data input.

For standalone software testing, an average of 1411 images were used (minimum 694, maximum 1528), with an average sensitivity of 91.8% (minimum 86.9, maximum 98.3), specificity of 91.5 (minimum 88.5, maximum 95.7), and AUC of 0.96 (minimum 0.95, maximum 0.98). For reader study testing, an average of 274 images were used for the evaluation (minimum 240, maximum 390), with an average sensitivity of 80.4% (minimum 75.9, maximum 85), specificity of 47.4% (minimum 25.8, maximum 69), and AUC of 0.84 (minimum 0.8, maximum 0.88). Currently, no AI/ML medical devices for breast cancer detection and diagnostics have been approved in Japan.

### SARS-Cov-2(COVID-19) detection

In Japan, three medical devices^68–70^ aimed at detecting COVID-19 infection have been approved based on standalone software testing. In response to the rapid spread of COVID-19, all devices were fast-tracked for evaluation and approved within two months of application. The average number of images used to evaluate the performance of these devices was 370.3 (minimum 190, maximum 704) with an average sensitivity and specificity of 84.9% (minimum 77.7, maximum 89.6) and 54.7 (minimum 37.1, maximum 90.7) respectively. AUC was not reported in any study.

## Discussion

In this study, we extracted AI/ML-based CAD devices approved in the USA and Japan and thoroughly assessed the performance evaluation methods. The main findings are as follows: (1) In the USA, devices classified as CAST were approved based on standalone software testing, and all devices classified as CADe/CADx were approved based on reader study testing. However, in Japan, there is no clear classification. (2) AI/ML-based CAD in the field of endoscopy for the detection of colorectal polyps was approved based on the data of a prospective RCT in the USA. In Japan, it was approved based on the evaluation of the software alone. This difference was influenced by the fact that the use of colonoscopy in the medical healthcare system in the two countries is quite different, as discussed in “Necessity of prospective testing” section. (3) In the USA, a wider variety of devices are available, compared to the devices available in Japan. To the best of our knowledge, this is the first comprehensive systematic comparative analysis of evaluation methods for AL/ML-based CAD devices approved in the USA and Japan.

### Different methodological approaches to standalone software testing and a reader study testing

There are two major testing methods for evaluating AI/ML-based CAD devices: standalone software testing and reader study testing. The 31 devices approved as CAST in the USA were all evaluated by software alone. On the other hand, all the devices classified as CADe/CADx were subjected to reader study testing, except for post-market improvements.

CAST is said to have been introduced by Goldenberg et al. in 2011^4,71^. In the USA, devices classified as having CAST functions are intended for use in urgent situations, such as intracerebral hemorrhage or cerebrovascular obstruction, where the devices assist non-specialists in promptly determining the best course of action to take. As the devices may contribute to the clinical decision-making process, software test results are required to demonstrate sensitivity and specificity of 90% or higher. The mean values of sensitivity, specificity, and AUC for all approved CAST devices in the USA were high—92.9% (minimum 84.3, maximum 98.3), 91.7% (minimum 83.5, maximum 97.6), 0.96 (minimum 0.91, maximum 0.99), respectively.

The FDA has issued guidance on the standalone evaluation of software and recommends AUC, sensitivity, and specificity as evaluation indexes^72^. Probably owing to this guidance, these indices were evaluated for many devices. These evaluation indexes would contribute to a reasonable evaluation of the performances of newly developed devices.

Furthermore, in the USA, all devices were tested using the multiple reader multiple case (MRMC) method. The reported average number of doctors who participated in the test was 19.2 (minimum 14, maximum 24). Of the devices approved based on reader study testing, five were conducted using “Fully-Crossed design” following the FDA recommendation due to its greater statistical power. The test design is recommended by the FDA when the output results are displayed concurrently (2020 revision)^73^.

Despite the extensive testing procedures that were performed before approval, there were instances where the clinical performance of approved devices did not measure up to expectations^74–76^. This was the case with BriefCase for CSF Triage (Aidoc Medical Ltd.) and Health VCF (Zebra Medical Vision Ltd.). Such cases underline the necessity of analyzing the generalizations contained within the current evaluation methods for increasingly diverse devices.

There are currently no approved CAST devices in Japan; hence, it was not possible to find any information on an evaluation method for CAD devices that fall under this category. The data indicates that, in Japan, the method used to evaluate the performance of a device is not reliant on the category the device is classified by, be it CAST or CADe/CADx. We believe that the reason there were no PMDA-approved CAST devices in Japan lies in the medical environment differences when compared to the USA. For instance, according to data reported by the Organization for Economic Co-operation and Development in a survey on the distribution of medical equipment^77^, Japan ranked highest in the CT scanner category with 111 scanners for every 1,000,000 people; The USA ranked 11th with only 43 (27 in hospitals and 16 ambulatories) scanners per 1,000,000 people. However, the reported number of CT examinations per 1000 individuals for both countries was comparable: 200–250 exams per 1000 individuals. Thus, it is evident that, over an identical period, the data volume outputted by a single CT scanner in the USA would be far greater than that in Japan. Therefore, the need for prompt screening of high-risk patients may be greater in the USA than in Japan, which may be the reason why CAST devices are widely developed in the USA.

In Japan, there is no guidance on the evaluation of devices by standalone software testing or by reader study testing. Establishing such guidance along with evaluation indexes may be necessary if Japan hopes to continue promoting research and development of AI/ML-based medical devices.

### Necessity of prospective testing

Of the 57 AI/ML-based CAD devices selected and analyzed in the present study, three devices conducted prospective studies: IDx-DR (IDx) and EyeArt (Eyenuk, Inc), for the detection of diabetic retinopathy, and GI Genious (Cosmo Artificial Intelligence-AI, LTD) for the detection of colorectal polyps. The common denominator between these three devices is that the quality of the image used as input data into the software greatly depends on the skill of the surgeon. Hence, the performance of the AI/ML-based CAD device is considerably dependent on the dexterity of the user, and a less skilled professional may not be able to realize the full potential of the device. Moreover, the dependence of such devices on the user’s skills leads to a higher likelihood than some of their counterparts to misdiagnose. Although many studies have been reported on AI/ML-based CAD devices for ultrasonography used to detect breast tumors^78^, it has not yet been granted regulatory approval. It was speculated that this is because the imaging skill of the operator had a significant impact on the performance of the software.

We believe that the difference between CAD-assisted colorectal polyp detection in the USA and Japan is due to the significant differences in the clinical positioning of colonoscopy in the healthcare system.

In the USA, colonoscopy is recognized as the “gold-standard” screening test for colorectal cancer prevention. Most practitioners choose to remove all polyps found during a colonoscopy. Therefore, there is a concern that the use of AI/ML-based CAD devices will inevitably increase the number of polyps detected, including benign ones, thereby increasing the burden of the procedure on the patients. Furthermore, for colonoscopy, rather than sensitivity and specificity, the best indicator for performance evaluation is the adenoma detection rate (ADR)^79,80^. Indeed, the ADR index has been confirmed to be directly correlated with the mortality rate of colorectal cancer^81,82^.

In Japan, when a polyp is found during colonoscopy, the physician makes a qualitative diagnosis using a magnifying endoscope and makes a judgment on its malignancy level. The term “semi-clean colon” refers to a colon with a small adenoma judged as benign (also known as microadenomas, less than 5 mm wide), for which a follow-up is performed without excision^83^. This indicates that not all polyps are extracted during a procedure in Japan, contrary to the procedure in the USA. Therefore, the impact of colonoscopy on treatment strategy differs between Japan and the USA, which might have resulted in the approval of the CADe device based on the evaluation of the standalone software testing in Japan.

Furthermore, to evaluate the performance of the CAD system for detecting colorectal polyps, video frames were used as test data and the performance was evaluated in an actual clinical practice manner. It can be said that the PMDA has made a reasonable evaluation of CAD for colorectal polyp detection in line with the clinical scenario in Japan.

IDx-DR and EyeArt devices designed for the detection of diabetic retinopathy are used in primary care facilities. These devices, using the results of AI analysis, are intended to help caregivers decide whether to encourage patients to see specialists. These devices are intended to be operated by non-expert practitioners. Therefore, it is necessary for manufacturers to properly train operators to be able to use the device to achieve its full potential and create an appropriate imaging protocol. This explains why imageability was also used as an evaluation factor when reviewing the performance of such devices^84^.

### Comparison of diversity of AI/ML-based CAD

A comprehensive analysis of AI/ML-based CAD devices approved by the regulatory agencies revealed that the FDA approved a wider variety of devices than the PMDA. In the USA, AI/ML-based CAD for intracerebral hemorrhage, cerebrovascular obstruction, breast cancer, pneumothorax, pulmonary embolism, and pleural effusion diagnosis is a field that remains on the cutting edge of the healthcare industry. The constantly updated and improved head CT, mammogram, and chest CT databases may be one of the reasons for such technological advances. Indeed, digital databases such as the Digital Database for Screening Mammography (DDSM)^85^, or ChexPert^86^ are known for their large-scale database and are frequently used in studies on image analysis algorithm development. Furthermore, the National Institute of Health created the ChestNet-14^87^ dataset available through Kaggle (an online community periodically organizing data science competitions). ChestNet-14 comprises 112,000 images of 14 different types of lesions. Similarly, the Radiological Society of North America published 25,312 head CT images on Kaggle^88^. As can be seen from these instances, there is a constant push for the further development of AI/ML-based CAD-assisted diagnostic/detection devices.

Historically, the USA pioneered the application of CAD to the medical field, with the FDA approving the world’s first CAD device in 1998 (“Image Checker”^89^, by R2, now manufactured by Hologic). This is assumed to be one of the reasons why medical AI/ML research and development is at an advanced stage as compared to other countries.

The most advanced AI/ML-based CAD sector in Japan targets the colonoscopy market. Currently, the Japanese company, Olympus, accounts for 70% of the endoscopes’ global market shares^90^. It is speculated that this may be a factor for the use of AI in the analysis of endoscopic images, and lesion detection is more advanced as compared to the other areas. In Japan, research teams at Showa University and Nagoya University have published a database (SUN^91^) containing 49,799 colorectal polyps. Therefore, further research and development focusing on this area are required.

### Future work

Because the European Union’s European Medicines Agency (EMA) is another important regulatory agency, including AI/ML-based medical devices approved by the EMA would result in a more complete analysis of the current state of global device approval procedures. However, the EMA does not appear to have a comprehensive database accessible to the public. If the EMA makes its data publicly available, we will incorporate it in a future study, generating results of higher quality and consistency.

### Limitations

The present study had two limitations. First, the devices that were approved in the USA or Japan were extracted using their general names or product codes. Therefore, there may be relevant devices that we have not identified. Second, this systematic analysis was limited to AI/ML-based CAD devices. Nevertheless, we believe that our comprehensive analysis and comparison of evaluation methods of AL/ML-based medical devices in terms of target and study design between the USA and Japan provide valuable knowledge on the global development of AI/ML-based CAD.

## Conclusions

To the best of our knowledge, the present study is the first systematic comprehensive comparative analysis to clarify differences in performance evaluation methods of AI/ML-based CAD devices approved in the USA and Japan.

In the USA, there are two prevalent methods for performance evaluation: standalone software testing and reader study testing. Which one is used depends on whether the device is CAST or CADe/CADx. In contrast, Japan does not make such a clear distinction, as illustrated by the indiscriminate use of either standalone software testing or reader study testing for performance evaluation of devices belonging to the same class label (CADe/CADx). In addition to this, the present study indicated that the AI/ML CAD devices approved in the USA were much more diverse than those approved in Japan. As a regulatory agency, the FDA has issued clear guidance specifying and describing points to keep in mind when conducting standalone software testing or reader study testing. The authors believe that the active publication of such guidance and extensive comprehensive documentation by regulatory agencies encourages the development of AI/ML medical devices. Finally, from the perspective of mutual acceptance of AI/ML-based CAD devices developed in both countries, it would seem relevant to address the issue of international harmonization of AI/ML-based CAD evaluation to obtain consensus on reliable evaluation methods for these devices.

## Methods

### Extraction of AI/ML-based medical devices in the USA

AI/ML medical devices were extracted from the FDA product code database^11^. Product Code is a “3-character unique product identifier”. As of June 22, 2021 (the date at which devices were selected), 6701 product codes were listed. Two independent authors performed a keyword search and determined whether the AI-based CAD met the inclusion criteria and resolved discrepancies by joint review and consensus. When using search keywords such as “artificial intelligence,” “machine learning,” and “deep learning,” 18 product codes were identified (8 for artificial intelligence, 9 for machine learning, and 1 for deep learning). Among the 18 product codes, seven duplicates were removed, and five other product codes were excluded after screening (excluding codes that did not correspond to triage, notification, detection, or diagnosis). The final six product codes encompassed a total of 48 devices. Of these 48 devices, four were granted de novo clearance and 44 had been granted 510(k) clearance [no premarket approval (PMA)]. Two devices were excluded from this study because of insufficient information in the 510(k) summary; one was excluded because of minor changes in the target user. The final number of US-approved devices used in the present study was 45. Details of the screening and selection processes are shown in Fig. 1.

Information on de novo classification requests, decision summaries, and a 510(k) summary of AI/ML-based CAD approved in the USA was collected. Information on (1) device name; (2) manufacturer; (3) approval date; (4) intended use; (5) test method; (6) target disease; (7) test data volume; and (8) performance [sensitivity, specificity, area under the curve (AUC), and accuracy rate] was retrieved.

### Extraction of AI/ML-based medical devices in Japan

Japanese PMDA-approved AI/ML medical devices were extracted from the database of the Japan Association for the Advancement of Medical Equipment using its search service (JAAME Search)^12^. The JAAME database comprehensively stores the general names of medical devices and information on approved or certified medical devices. First, since this study focuses on AI/ML medical devices used to diagnose specific diseases, the initial search was performed using a “disease diagnostic program.” The search results showed 165 device categories with generic names (equivalent to FDA product codes). These 165 categories comprised a combined total of 349 approved/certified medical devices (as of June 22, 2021).

Since the first AI/ML medical device approved in Japan was EndoBRAIN (CYBERNET), for which approval was issued in December 2018^14^, the search was refined to devices approved between December 2018 and June 2021. This reduced the number of devices that matched all selected characteristics to 57. After excluding devices for genome analysis or other non-image-based tasks, 32 devices remained. The final data assessment checked whether using AI/ML from the press release information and package inserts of the devices, yielding 12 devices for study.

### Classification of AI/ML-based CAD

Identified AI/ML-based CAD devices were classified based on the definitions of CADe, CADx, and CAST described in the guidance document by the FDA^72^. Taking the example of lesion detection, a device that outputs the mark or emphasis is a CADe, a device that identifies the malignancy level of the lesion is a CADx, and a device whose output is meant to reduce or eliminate the burden of doctors is a CAST.

### Data analysis

After grouping the identified devices according to their target area, we further divided them into subgroups according to the evaluation method used for approval (standalone software testing or reader study testing). Known data averages for the test cases, sensitivity, specificity, and AUC results were calculated (minimum–maximum) using Microsoft Excel.

### Research involving human participants

This study is a systematic review and do not involve human participants.

## Data Availability

The authors declare that all the data included in this study are available within the paper.

## References

[1] Benjamens S, Dhunnoo P, Meskó B (2020). The state of artificial intelligence-based FDA-approved medical devices and algorithms: An online database. NPJ. Digit. Med..

[2] Muehlematter UJ, Daniore P, Vokinger KN (2021). Approval of artificial intelligence and machine learning-based medical devices in the USA and Europe (2015–20): A comparative analysis. Lancet Digit. Health.

[3] Allen B, Agarwal S, Coombs L, Wald C, Dreyer K (2020). ACR data science institute artificial intelligence survey. J. Am. Coll. Radiol..

[4] Goldenberg R, Peled N (2011). Computer-aided simple triage. Int. J. Comput. Assist. Radiol. Surg..

[5] Kohli A, Mahajan V, Seals K, Kohli A, Jha S (2019). Concepts in U.S. Food and Drug Administration regulation of artificial intelligence for medical imaging. AJR Am. J. Roentgenol..

[6] Ferryman K (2020). Addressing health disparities in the Food and Drug Administration's artificial intelligence and machine learning regulatory framework. J. Am. Med. Inform. Assoc..

[7] Hernandez-Boussard T, Lundgren MP, Shah N (2021). Conflicting information from the Food and Drug Administration: Missed opportunity to lead standards for safe and effective medical artificial intelligence solutions. J. Am. Med. Inform. Assoc..

[8] Lin H (2019). Diagnostic efficacy and therapeutic decision-making capacity of an artificial intelligence platform for childhood cataracts in eye clinics: A multicentre randomized controlled trial. EClinicalMedicine.

[9] Calisto FM, Santiago C, Nunes N, Nascimento JC (2022). BreastScreening-AI: Evaluating medical intelligent agents for human-AI interactions. Artif. Intell. Med..

[10] Calisto FM, Santiago C, Nunes N, Nascimento JC (2021). Introduction of human-centric AI assistant to aid radiologists for multimodal breast image classification. Int. J. Hum. Comput..

[11] FDA. Product Code Classification Database. https://www.fda.gov/medical-devices/classify-your-medical-device/product-code-classification-database.

[12] JAAME. Search. http://www.jaame.or.jp/.

[13] Repici A (2020). Efficacy of real-time computer-aided detection of colorectal neoplasia in a randomized trial. Gastroenterology.

[14] CYBERNET. EndoBRAIN. https://www.pmda.go.jp/PmdaSearch/kikiDetail/ResultDataSetPDF/331289_23000BZX00372000_1_01_01 (2020).

[15] CYBERNET. EndoBRAIN-UC. https://www.pmda.go.jp/PmdaSearch/kikiDetail/ResultDataSetPDF/331289_30200BZX00136000_1_01_01 (2020).

[16] CYBERNET. EndoBRAIN-EYE. https://www.pmda.go.jp/PmdaSearch/kikiDetail/ResultDataSetPDF/331289_30200BZX00208000_1_01_01 (2021).

[17] CYBERNET. EndoBRAIN-Plus. https://www.pmda.go.jp/PmdaSearch/kikiDetail/ResultDataSetPDF/331289_30200BZX00235000_1_01_01 (2020).

[18] NEC. WISE VISION. https://www.pmda.go.jp/PmdaSearch/kikiDetail/ResultDataSetPDF/581038_30200BZX00382000_A_01_01 (2020).

[19] FUJIFILM. EW10-EC02. https://www.pmda.go.jp/PmdaSearch/kikiDetail/ResultDataSetPDF/671001_30200BZX00288000_A_01_02 (2020).

[20] Weigt J (2021). Performance of a new integrated computer-assisted system (CADe/CADx) for detection and characterization of colorectal neoplasia. Endoscopy.

[21] FDA. 510(k) Summary for Briefcase for PE. https://www.accessdata.fda.gov/cdrh_docs/pdf19/K190072.pdf (2019).

[22] FDA. 510(k) Summary for HealthPNX. https://www.accessdata.fda.gov/cdrh_docs/pdf19/K190362.pdf (2019).

[23] FDA. 510(k) Summary for Critical Care Suite. https://www.accessdata.fda.gov/cdrh_docs/pdf18/K183182.pdf (2019).

[24] FDA. 510(k) Summary for HealthCXR. https://www.accessdata.fda.gov/cdrh_docs/pdf19/K192320.pdf (2019).

[25] FDA. 510(k) Summary for red dot. https://www.accessdata.fda.gov/cdrh_docs/pdf19/K191556.pdf (2020).

[26] FDA. 510(k) Summary for AIMI-Triage CXR PTX. https://www.accessdata.fda.gov/cdrh_docs/pdf19/K193300.pdf (2020).

[27] FDA. 510(k) Summary for BriefCase for iPE Triage. https://www.accessdata.fda.gov/cdrh_docs/pdf20/K201020.pdf (2020).

[28] FDA. 510(k) Summary for CINA CHEST. https://www.accessdata.fda.gov/cdrh_docs/pdf21/K210237.pdf (2020).

[29] FDA. 510(k) Summary for BriefCase for Free Gas. https://www.accessdata.fda.gov/cdrh_docs/pdf19/K193298.pdf (2020).

[30] FUJIFILM. FS-AI688. https://www.pmda.go.jp/PmdaSearch/kikiDetail/ResultDataSetPDF/671001_30200BZX00150000_A_01_03 (2020).

[31] LPIXEL. EIRL X-ray Lung nodule. https://www.pmda.go.jp/PmdaSearch/kikiDetail/ResultDataSetPDF/171955_30200BZX00269000_B_00_02 (2020).

[32] FDA. Evaluation of automatic class III designation for contact decision summary. https://www.accessdata.fda.gov/cdrh_docs/reviews/DEN170073.pdf (2018).

[33] FDA. 510(k) Summary for BriefCase for ICH https://www.accessdata.fda.gov/cdrh_docs/pdf18/K180647.pdf (2018).

[34] FDA. 510(k) Summary for Accipiolx. https://www.accessdata.fda.gov/cdrh_docs/pdf18/K182177.pdf (2018).

[35] FDA. 510(k) Summary for HealthICH. https://www.accessdata.fda.gov/cdrh_docs/pdf19/K190424.pdf (2019).

[36] FDA. 510(k) Summary for DeepCT. https://www.accessdata.fda.gov/cdrh_docs/pdf18/K182875.pdf (2019).

[37] FDA. 510(k) Summary for BriefCase for LVO. https://www.accessdata.fda.gov/cdrh_docs/pdf19/K192383.pdf (2019).

[38] FDA. 510(k) Summary for Viz ICH. https://www.accessdata.fda.gov/cdrh_docs/pdf19/K193658.pdf (2020).

[39] FDA. 510(k) Summary for RAPID ICH. https://www.accessdata.fda.gov/cdrh_docs/pdf19/K193087.pdf (2020).

[40] FDA. 510(k) Summary for CuraRad-ICH. https://www.accessdata.fda.gov/cdrh_docs/pdf19/K192167.pdf (2020).

[41] FDA. 510(k) Summary for NinesAI. https://www.accessdata.fda.gov/cdrh_docs/pdf19/K193351.pdf (2020).

[42] FDA. 510(k) Summary for qER. https://www.accessdata.fda.gov/cdrh_docs/pdf20/K200921.pdf (2020).

[43] FDA. 510(k) Summary for CINA. https://www.accessdata.fda.gov/cdrh_docs/pdf20/K200855.pdf (2020).

[44] FDA. 510(k) Summary for Rapid LVO 1.0. https://www.accessdata.fda.gov/cdrh_docs/pdf20/K200941.pdf (2020).

[45] FDA. 510(k) Summary for Accipiolx. https://www.accessdata.fda.gov/cdrh_docs/pdf20/K201310.pdf (2020).

[46] FDA. 510(k) Summary for HALO. https://www.accessdata.fda.gov/cdrh_docs/pdf20/K200873.pdf (2020).

[47] FDA. 510(k) Summary for Viz ICH. https://www.accessdata.fda.gov/cdrh_docs/pdf21/K210209.pdf (2021).

[48] LPIXEL. EIRL aneurysm. https://www.info.pmda.go.jp/downfiles/md/PDF/171955/171955_30100BZX00142000_A_00_03.pdf (2019).

[49] FDA. DE NOVO CLASSIFICATION REQUEST FOR IDx-DR. https://www.accessdata.fda.gov/cdrh_docs/reviews/DEN180001.pdf (2018).

[50] FDA. 510(k) Summary for EyeArt. https://www.accessdata.fda.gov/cdrh_docs/pdf20/K200667.pdf (2020).

[51] FDA. 510(k) Summary for IDx-DR. https://www.accessdata.fda.gov/cdrh_docs/pdf20/K203629.pdf (2021).

[52] FDA. Evaluation of automatic class III designation for osteodetect. https://www.accessdata.fda.gov/cdrh_docs/reviews/DEN180005.pdf (2018).

[53] FDA. 510(k) Summary for FractureDetect. https://www.accessdata.fda.gov/cdrh_docs/pdf19/K193417.pdf (2020).

[54] FDA. 510(k) Summary for BriefCase for C-spine. https://www.accessdata.fda.gov/cdrh_docs/pdf19/K190896.pdf (2019).

[55] FDA. 510(k) Summary for HealthVCF. https://www.accessdata.fda.gov/cdrh_docs/pdf19/K192901.pdf (2020).

[56] FDA. 510(k) Summary for uAI EasyTriage-Rib. https://www.accessdata.fda.gov/cdrh_docs/pdf19/K193271.pdf (2021).

[57] FDA. 510(k) Summary for cmTriage. https://www.accessdata.fda.gov/cdrh_docs/pdf18/K183285.pdf (2019).

[58] FDA. 510(k) Summary for ProFound AI Software V2.1. https://www.accessdata.fda.gov/cdrh_docs/pdf19/K191994.pdf (2019).

[59] FDA. 510(k) Summary for Transpara, https://www.accessdata.fda.gov/cdrh_docs/pdf18/K181704.pdf. (2018).

[60] FDA. 510(k) Summary for Transpara 1.5. https://www.accessdata.fda.gov/cdrh_docs/pdf19/K192287.pdf (2019).

[61] FDA. 510(k) Summary for Transpara 1.6. https://www.accessdata.fda.gov/cdrh_docs/pdf19/K193229.pdf (2020).

[62] FDA. 510(k) Summary for Transpara 1.7. https://www.accessdata.fda.gov/cdrh_docs/pdf21/K210404.pdf (2021).

[63] FDA. 510(k) Summary for HealthMammo. https://www.accessdata.fda.gov/cdrh_docs/pdf20/K200905.pdf (2020).

[64] FDA. 510(k) Summary for Saige-Q. https://www.accessdata.fda.gov/cdrh_docs/pdf20/K203517.pdf (2021).

[65] FDA. 510(k) Summary for PowerLook Tomo Detection V2 Software. https://www.accessdata.fda.gov/cdrh_docs/pdf18/K182373.pdf (2018).

[66] FDA. 510(k) Summary for MammoScreen. https://www.accessdata.fda.gov/cdrh_docs/pdf19/K192854.pdf (2020).

[67] FDA. 510(k) Summary for Genius AI Detection. https://www.accessdata.fda.gov/cdrh_docs/pdf20/K201019.pdf (2020).

[68] CESdecartes. InferRead CT Pneumonia. https://www.pmda.go.jp/files/000235941.pdf (2020).

[69] Corp, M. M. Ali-M3. https://www.pmda.go.jp/files/000235943.pdf (2020).

[70] FUJIFILM. FS-AI693. https://www.pmda.go.jp/PmdaSearch/kikiDetail/ResultDataSetPDF/671001_30300BZX00145000_A_01_01 (2021).

[71] Goldenberg R (2012). Computer-aided simple triage (CAST) for coronary CT angiography (CCTA). Int. J. Comput. Assist. Radiol. Surg..

[72] FDA. Computer assisted detection devices applied to radiology images and radiology device data. https://www.fda.gov/media/77635/download (2012).

[73] FDA. Clinical performance asessment considerations for computer-assisted detection devices applied to radiology images and radiology device data. https://www.fda.gov/media/77642/download (2020).

[74] Small JE, Osler P, Paul AB, Kunst M (2021). CT cervical spine fracture detection using a convolutional neural network. AJNR Am. J. Neuroradiol..

[75] Voter AF, Larson ME, Garrett JW, Yu JJ (2021). Diagnostic accuracy and failure mode analysis of a deep learning algorithm for the detection of cervical spine fractures. AJNR Am. J. Neuroradiol..

[76] Kolanu N (2020). Clinical utility of computer-aided diagnosis of vertebral fractures from computed tomography images. J. Bone Miner. Res..

[77] OECD. Health Statistics 2019 Frequently Requested Data. https://www.oecd.org/els/health-systems/health-data.htm (2019).

[78] Aggarwal R (2021). Diagnostic accuracy of deep learning in medical imaging: a systematic review and meta-analysis. NPJ Digit. Med..

[79] Abdelfatah MM, Elhanafi S, Zuckerman MJ, Othman MO (2017). Correlation between adenoma detection rate and novel quality indicators for screening colonoscopy. A proposal for quality measures tool kit. Scand. J. Gastroenterol..

[80] Lee TJ (2012). Colonoscopy quality measures: Experience from the NHS Bowel Cancer Screening Programme. Gut.

[81] Kaminski MF (2017). Increased rate of adenoma detection associates with reduced risk of colorectal cancer and death. Gastroenterology.

[82] Meester RG (2015). Variation in adenoma detection rate and the lifetime benefits and cost of colorectal cancer screening: A microsimulation model. JAMA.

[83] Lieberman DA (2012). Guidelines for colonoscopy surveillance after screening and polypectomy: A consensus update by the US Multi-Society Task Force on Colorectal Cancer. Gastroenterology.

[84] Abramoff MD, Lavin PT, Birch M, Shah N, Folk JC (2018). Pivotal trial of an autonomous AI-based diagnostic system for detection of diabetic retinopathy in primary care offices. NPJ Digit. Med..

[85] Sun L (2021). Breast mass detection in mammography based on image template matching and CNN. Sensors (Basel).

[86] Irvin, J. *et al.* CheXpert: A large chest radiograph dataset with uncertainty labels and expert comparison. http://arxiv.org/abs/1901.07031 (2019).

[87] Rajpurkar, P. *et al.* CheXNet: Radiologist-level pneumonia detection on chest X-rays with deep learning. https://arxiv.org/abs/1711.05225v3 (2017).

[88] Flanders AE (2020). Construction of a machine learning dataset through collaboration: The RSNA 2019 Brain CT Hemorrhage Challenge. Radiol. Artif. Intell..

[89] HOLOGIC. ImageChecker2D CAD Technology (accessed 3 January 2022). https://www.3dimensionsmammography.eu/screening-portfolio/imagechecker-2d-cad-technology/#.

[90] CreditSuiss. Olympus. https://research-doc.credit-suisse.com/docView?sourceid=em&document_id=x723296&serialid=W7IBkVKcu8%2bhr5IOVulyTtnDvAUx6q9n976C6C%2bkc08%3d (2016).

[91] Brown JRG, Berzin TM (2021). EndoBRAIN-EYE and the SUN database: Important steps forward for computer-aided polyp detection. Gastrointest. Endosc..

